# The structural reserve of brain networks influences outcomes after a stroke

**DOI:** 10.1093/braincomms/fcaf456

**Published:** 2025-11-20

**Authors:** Lukas Frontzkowski, Tim J Hunze, Winifried Backhaus, Marlene Bönstrup, Christian Gerloff, Bastian Cheng, Götz Thomalla, Benedikt M Frey, Paweł P Wróbel, Hanna Braaß, Philipp J Koch, Focko L Higgen, Fanny Quandt, Robert Schulz

**Affiliations:** Department of Neurology, University Medical Center Hamburg-Eppendorf, Hamburg 20251, Germany; Department of Nuclear Medicine, University Hospital, LMU, Munich 81377, Germany; Institute for Stroke and Dementia Research (ISD), LMU, Munich 81377, Germany; Department of Neurology, University Medical Center Hamburg-Eppendorf, Hamburg 20251, Germany; Department of Neurology, University Medical Center Hamburg-Eppendorf, Hamburg 20251, Germany; Department of Neurology, University Medical Center Hamburg-Eppendorf, Hamburg 20251, Germany; Department of Neurology, University Medical Center Frankfurt, Frankfurt 60528, Germany; Department of Neurology, University Medical Center Hamburg-Eppendorf, Hamburg 20251, Germany; Department of Neurology, University Medical Center Hamburg-Eppendorf, Hamburg 20251, Germany; Department of Neurology, University Medical Center Hamburg-Eppendorf, Hamburg 20251, Germany; Department of Neurology, University Medical Center Hamburg-Eppendorf, Hamburg 20251, Germany; Department of Neurology, University Medical Center Hamburg-Eppendorf, Hamburg 20251, Germany; Department of Neurology, University Medical Center Hamburg-Eppendorf, Hamburg 20251, Germany; Department of Neurology, University Medical Center Lübeck, Lübeck 23562, Germany; Department of Neurology, University Medical Center Hamburg-Eppendorf, Hamburg 20251, Germany; Department of Neurology, University Medical Center Hamburg-Eppendorf, Hamburg 20251, Germany; Department of Neurology, University Medical Center Hamburg-Eppendorf, Hamburg 20251, Germany

**Keywords:** stroke, connectome, graph theory, recovery, rehabilitation

## Abstract

Structural brain reserve capacity has recently gained an increasing interest in stroke recovery research. Focal and global measures of brain reserve have been linked with recovery trajectories. Whether the reserve localized within large-scale brain networks might also carry information to better understand outcome variability after stroke is an open question. This work analysed 31 patients with severe, first-ever unilateral, supratentorial stroke. Patients underwent MRI brain imaging and clinical testing within the first 2 weeks after the event and a longitudinal clinical follow-up after 3–6 months. Individual tractography in the contralesional hemisphere was performed to reconstruct structural connectomes to approximate the state of the ipsilesional brain networks before the stroke. Graph theory was applied to describe network integration and segregation topologies. Linear and ordinal logistic regression analyses were conducted to associate network topologies at baseline with neurological symptom burden, global and activity-related disability and motor impairment at follow-up. The main finding was that less segregated and more integrated networks, characterized by lower network modularity and higher efficiency, were linked with a more favourable outcome on follow-up. Modularity exerted a remarkably consistent influence across various outcome measures. This association was independent of the initial deficit, lesion volume or age. This study sheds novel light on brain reserve, localizing within the topology of pre-stroke structural brain networks, as a critical determinant of recovery after stroke.

## Teaser

Frontzkowski and Hunze *et al*. show that differences in brain network architecture influence stroke recovery. Patients with more efficient and less modular structural networks in the contralesional hemisphere experienced more favourable long-term outcomes, emphasizing the importance of brain network reserve as a critical determinant of recovery after stroke.

## Graphical Abstract

**Graphical Abstract:**
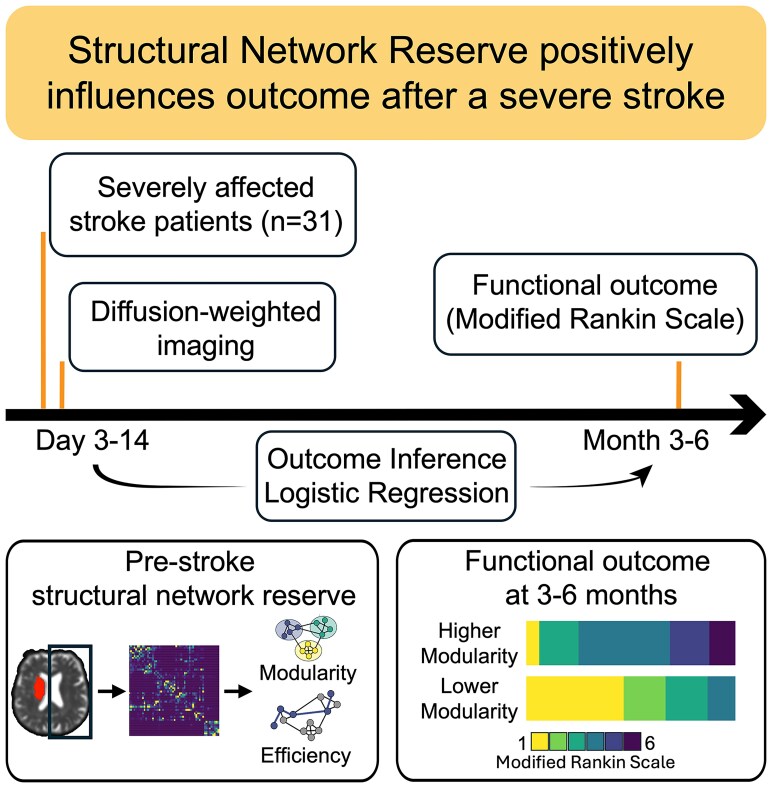

## Introduction

Brain reserve capacity has become increasingly recognized as a critical, influential factor for rehabilitation trajectories and outcomes after ischaemic stroke.^1,2^ Aside from cognitive reserve, structural brain reserve has gained particular interest, as neuroimaging offers an objective way to characterize brain parameters in individual patients and link them to their specific recovery trajectory. Various parameters of structural brain reserve, i.e. surrogates of pre-stroke states of the brain, have been developed towards informative biomarkers to deepen our mechanistic understanding of recovery processes after stroke and improve predictive outcome models in large clinical cohorts. Global measures include relative brain age,^3^ white matter hyperintensity burden or the degree of brain atrophy.^4,5^ More recently, studies have focused on relatively focal measures of brain reserve, such as those attributed to the cerebellum^6^ or dopaminergic mesolimbic brain regions.^7^

Network neuroscience has made significant contributions to stroke recovery research in recent years. It has extended the focus of previous analyses, which have primarily addressed isolated pathways such as the corticospinal tract or corticocortical connections,^8^ towards large-scale structural brain networks.^9,10^ Graph-theoretical analyses have provided a mathematical framework for modelling and quantifying the complex architecture of brain networks. In this framework, brain regions are represented as nodes, and their structural or functional connections are represented as edges, enabling a systematic analysis of the network topology.^11-15^ Various topological metrics of networks that estimate their functionality to integrate information processing across and segregate processing between brain areas, with aspects of modular processing, have been developed.^11,14^ In a healthy brain, these properties are well-tuned, enabling optimal information processing. After a stroke, the integrity and topology of the network become critically altered. Previous studies have shown that network alterations after a stroke show time- and recovery-dependent changes,^16^ can inform, quantified in the acute stage after stroke, about future recovery trajectories^9^ and even explain behavioural effects of acute stroke treatment such as thrombolysis.^17^ Despite relevant methodological differences, these structural analyses align with various functional network studies.^18,19^ Both approaches have evidenced that a stroke leads to less integrated and more segregated networks, directly accessible by specific graph measures that are, amongst others, global efficiency (GE) and modularity, respectively.^13^ Along with the concepts of brain reserve, we hypothesized that the pre-stroke state of large-scale structural brain networks would determine stroke recovery. We hypothesized that more integrated and less segregated networks before stroke would create an additional brain reserve, leading to better outcomes. When assessed in the first days after stroke, contralesional structural network topologies might render a reasonable proxy for their ipsilesional counterparts, opening an intriguing window into the pre-stroke state of the ipsilesional networks and their functionality in integration and segregation.

## Materials and methods

### Participants and clinical assessment

Patient data in this study is based on two previously published independent cohorts of acute ischaemic stroke patients. For the first cohort (C_1_), we initially recruited 61 patients from June 2012 to September 2017 who were admitted to the University Medical Center Hamburg-Eppendorf.^16,20^ For the second cohort (C_2_), 30 patients with initial severe impairment admitted to the same medical centre from October 2017 to February 2020 were initially recruited.^21^ Inclusion criteria for both studies were age ≥18 years; first-ever unilateral ischaemic stroke; a persistent motor deficit of the upper extremity, including hand function; and no history of severe psychiatric or neurological disease. Patients underwent brain imaging early after the stroke at time point T_1_ (C_1_, Days 3–5; C_2_, Days 3–14). Follow-up clinical data at time point T_2_ was in the late subacute stage after 3 months. A subset of 15 patients from cohort C_1_ was included in this work in which the Barthel index (BI) was ≤ 30 or the modified Rankin scale (mRS) was > 3 in the acute stage (Days 3–5). In cohort C_2_, 16 patients could be included in this work after excluding patients with missing or insufficient clinical or imaging data. This approach of cohort integration was successfully applied in our previous studies.^6^ For seven patients of cohort C_2_, follow-up data was gathered after 6 months due to unavailability at 3 months post-stroke. Follow-up clinical assessment included the mRS for global disability, the National Institutes of Health Stroke Scale (NIHSS) for neurological symptom burden, the BI for activity-related disability and the upper extremity score of the Fugl–Meyer assessment (UEFM) for motor impairment. In addition, 42 healthy controls (HC) were included. All participants provided informed consent or via a legal guardian, following the ethical Declaration of Helsinki. Original studies were granted permission by the local ethics committee of the Chamber of Physicians Hamburg (PV3777, PV5442 and PV5357).

### Image acquisition

Brain imaging for both datasets utilized a 3T Skyra MRI scanner (Siemens Healthineers, Erlangen, Germany). Employing a 32-channel head coil, the imaging sessions captured high-resolution T1- and T2-weighted images along with diffusion-weighted images (DWI). T1-weighted images were acquired using a three-dimensional magnetization-prepared rapid gradient echo sequence with the following parameters: repetition time (TR) = 2500 ms, echo time (TE) = 2.12 ms, flip angle 9°, encompassing 256 coronal slices with a voxel size of 0.8 × 0.8 × 0.9 mm³ and a field of view (FOV) of 240 mm. T2-weighted images were obtained through a fluid-attenuated inversion recovery sequence (TR = 9000 ms, TE = 86 ms, TI = 2500 ms, flip angle 150°, covering 43 transversal slices with a voxel size of 0.7 × 0.7 × 3.0 mm³ and a FOV of 230 mm). DWI utilized an echo planar imaging sequence covering the entire brain with gradients (*b* = 1500 s/mm²) applied along 64 non-collinear directions (TR = 10 000 ms, TE = 82 ms, flip angle 90°, 75 axial slices with a voxel size of 2 × 2 × 2 mm³, FOV of 256 mm). Additionally, a single b0 image was acquired.

### Image processing and analysis

Image processing has been described in detail in our previous work.^9^ In brief, we identified individual stroke lesions using a semi-automatic algorithm in ITK-SNAP (version 3.8.032) based on the visual analysis of T1-, T2- and diffusion-weighted images. Raw MRI data was preprocessed and reconstructed using QSIPrep (version 0.9.0.33). Resulting outputs were used to reconstruct whole-brain white matter fibre tracts via QSIPrep’s (matrix *single-shell_ss3t*) preconfigured workflow. Ultimately, the calculated weights of the white matter fibre tracts, set by the count of connecting streamlines, were included in the structural connectivity matrix based on the parcellation of the automated anatomical labelling (*AAL116*) atlas.^22^ The *AAL116* atlas consists of 58 regions per hemisphere, of which 41 are cortical, four are subcortical, and 13 are cerebellar. After excluding infratentorial and ipsilesional areas, the final contralesional structural connectivity matrices had a dimension of 45 × 45. We did not apply thresholds to structural connectomes,^23^ which aligns with our previous report.^9^ For sensitivity analyses, the Brainnetome atlas^24^ (matrix 123 × 123 after exclusion of infratentorial and ipsilesional areas) was also analysed as an alternative, with a more fine-grained subcortical segmentation and cortical parcellation.

### Structural network properties

**Figure 1:**
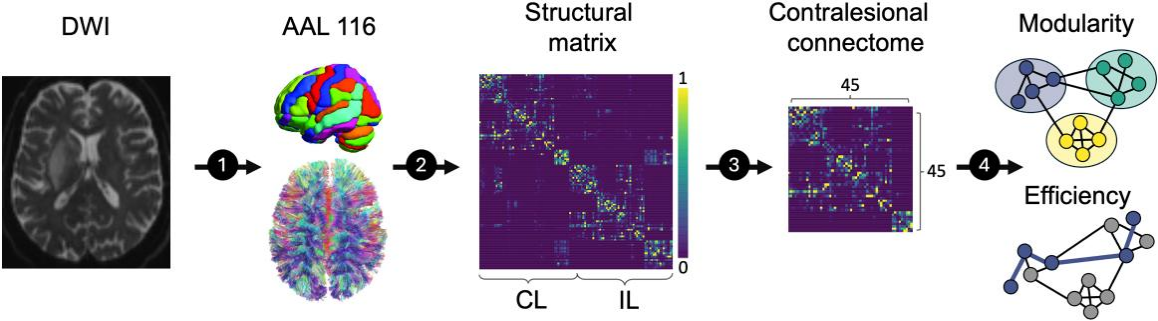
**Analysis steps.** First, DWI and T_2_-weighted images were preprocessed to compute structural connectomes based on the total fibre count via QSIPrep. Connectomes were parcellated according to the *AAL116* atlas to construct structural networks. Subsequently, ipsilesional and infratentorial regions of interest were removed, which resulted in 45 × 45 matrices. The Brain Connectivity Toolbox was applied to compute modularity and global efficiency. The contralesional connectome was analysed as a proxy for the pre-stroke state of the ipsilesional network. CL, contralesional; IL, ipsilesional.

Structural network properties were analysed using R (Version 4.4.1), MATLAB (R2023a) and the Brain Connectivity Toolbox.^15^ Weighted, undirected and normalized (connection weights between 0 and 1 depending on individual maximum) structural connectivity matrices were used for calculation. As suggested, we set self-connections to zero (brain-connectivity-toolbox.net). In line with our previous report, additional normalizations of the connectivity matrices were not carried out.^9^ Network topology was described by two established markers: modularity (MOD) and global efficiency (GE).^9,16^ MOD is a marker of network segregation. It represents the degree to which a community can be subdivided into non-overlapping modules (groups of nodes) so that there are a maximum number of within-module connections and a minimum number of between-module connections.^25^ GE, on the other hand, is a marker for network integration. It is the inverse of the average shortest path length across all network nodes.^26^ Evidence suggests that nodes and edges are not randomly arranged in the human connectome.^13^ Human brains tend to be organized in distinct modules that support functional segregation between specialized areas and interconnecting fibres, allowing for functional integration between these areas.^14^ Figure 1 gives an outline of the major analysis steps.

### Statistical analysis

Statistical analyses were conducted using R version 4.4.1.^27^ To examine the association of structural network properties (derived from imaging at time point T_1_) and clinical scores at T_2_, we computed ordinal logistic regression models (function *polr* from the MASS package) for mRS as the dependent variable of interest and separate linear regression models for NIHSS, BI and UEFM, respectively.^28^ In mRS models, MOD and GE values were considered after binary dichotomization to increase the interpretability and robustness of the models with more stable estimates given the small sample size. Following a median split, the patients were assigned to low and high MOD and GE groups. An odd number of patients (*n* = 31) led to a true median, which was then included in the low groups. Our previous studies have repeatedly used this approach.^6^ For linear models, MOD and GE were treated as continuous independent variables. All models treated age, lesion volume (log10-transformed to improve data distribution) and the initial neurological symptom burden (NIHSS) at T1 as independent variables. As age and network topologies show relevant collinearities,^29^ age was included after linear residualization against MOD/GE.^6^ From ordinal logistic regression models, we inferred odds ratios (OR) to score one level higher on mRS at T_2_. OR values below 1 would indicate a reduced risk of a worse outcome in patients exhibiting higher MOD or GE values. From linear models, standardized coefficients (Beta) were derived. Statistical significance was set at *P* < 0.05 (uncorrected).

## Results

### Demographic, clinical and lesion data

**Figure 2:**
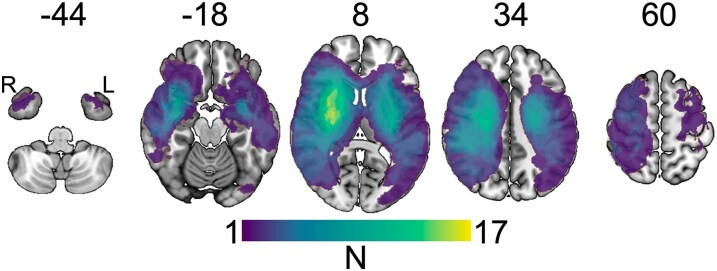
**Lesion heatmap.** The number of patients with lesioned voxels is colour-coded, with yellow indicating a high overlap between lesions. The numbers above the maps indicate the *z*-value (slice) in Montreal Neurological Institute (MNI) standard space. R, right; L, left; N, number.

Table 1 shows the individual clinical and demographic data. We investigated 31 severely affected stroke patients (16 females, 19 right-sided strokes, age 71.1 ± 11.7 years, mean ± SD, all supratentorial strokes). The median initial NIHSS score at T_1_ was 10 (interquartile range, IQR, 7–13), median mRS at T_2_ was 3 (IQR 1.5–4), median NIHSS at T_2_ was 3 (IQR 1.75–5.25), median BI at T_2_ was 85 (IQR 57.5–100), median UEFM at T_2_ was 31 (7–62) and lesion volume at T_1_ was 63.2 ± 75.0 ml (mean ± SD). Figure 2 gives a lesion heatmap.

**Table 1:** Demographic and clinical data

ID	Cohort	Age	Sex	Days of imaging	Side/Dom.	LV (ml)	mRS		NIHSS		BI	UEFM
							T_1_	T_2_	T_1_	T_2_	T_2_	T_2_
1	C1	81	M	3	L/d	1.7	4	3	4	2	85	65
2	C1	48	M	2	L/d	24.4	4	2	7	1	100	65
3	C1	87	F	3	L/d	1.0	4	1	1	0	100	64
4	C1	49	F	6	L/d	53.8	5	2	10	6	90	13
5	C1	73	F	4	L/d	5.8	4	4	9	3	50	13
6	C1	65	M	6	L/n	6.6	4	3	8	4	100	15
7	C1	69	M	4	R/d	25.1	4	1	3	1	100	62
8	C1	73	F	3	R/n	26.8	4	1	3	0	100	65
9	C1	70	F	6	R/n	74.4	4	1	5	2	95	58
10	C1	50	M	3	R/n	50.1	4	1	4		100	65
11	C1	43	M	4	R/n	79.8	4	2	13	3	100	16
12	C1	56	M	3	R/n	2.5	4	4	13	5	85	7
13	C1	77	F	3	R/n	9.1	5	3	8	2	85	
14	C1	85	F	6	R/n	16.7	4	4	7	4	65	40
15	C1	59	M	4	R/n	14.3	4	4	7			
16	C2	76	M	6	R/n	101.0	5	3	11			
17	C2	77	F	7	R/n	286.7	4	4^a^	11	10^a^	45^a^	4^a^
18	C2	71	F	8	R/n	38.4	5	3^a^	9	0^a^	70^a^	47^a^
19	C2	67	F	7	R/n	7.4	4	1^a^	11	7^a^	70^a^	5^a^
20	C2	80	M	7	R/n	108.4	5	6	16			
21	C2	79	F	6	R/n	120.4	5	4^a^	8	2^a^	85^a^	51^a^
22	C2	85	F	5	R/n	33.5	5	5	15	14	0	4
23	C2	78	M	4	R/n	178.1	5	4	17	3	65	15
24	C2	76	M	5	R/n	91.8	5	4	15	13	20	4
25	C2	73	F	5	R/n	27.6	4	1	5	2	80	62
26	C2	78	M	14	L/d	58.1	5	5^a^	17		15^a^	
27	C2	83	F	8	L/d	101.4	5	6	20			
28	C2	63	M	3	L/d	55.8	4	1	13	1	100	36
29	C2	80	F	12	L/d	20.5	5	4^a^	11	15^a^	30^a^	4^a^
30	C2	78	F	7	L/d	33.6	4	3	10	3	80	31
31	C2	74	M	10	L/d	303.3	5	5^a^	24		5^a^	4^a^

Clinical testing took place in the acute stage (T_1_), 3–14 days after the event and in the late subacute stage (T2). ^a^Either 3 or 6 months after stroke. M, male; F, female; Side, lesion side; Dom., Dominance (d, dominant hemisphere, n, non-dominant hemisphere); R, right; L, left; d, dominant hemisphere; n, non-dominant hemisphere; LV, lesion volume; mRS, modified Rankin scale; NIHSS, National Institutes of Health Stroke Scale; BI, Barthel index; UEFM, upper extremity score of the Fugl–Meyer assessment.

### Structural network topologies and recovery after stroke

**Figure 3:**
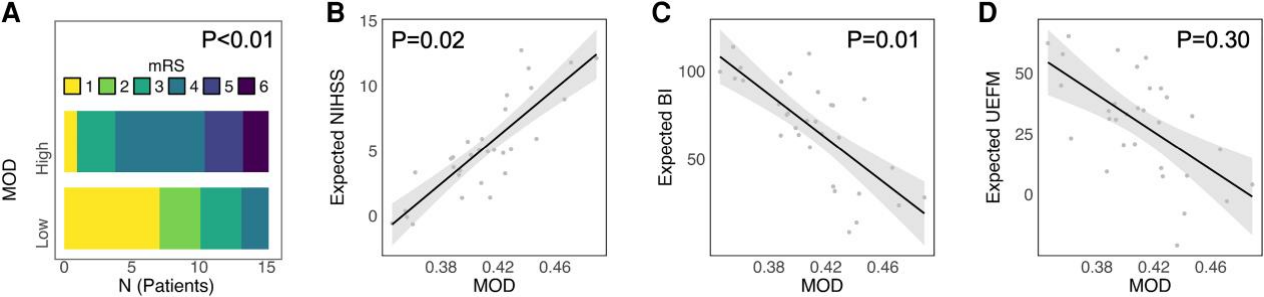
**Outcome correlation with network modularity.** MRS distribution is illustrated by stacked histograms (**A**) for high or low MOD of the structural network after median split dichotomization. For NIHSS (**B**), BI (**C**) and UEFM (**D**), effect plots are given for linear regression analyses with MOD with linear fit (grey line), 95% confidence intervals (shaded) and individual point estimates. Statistical analyses were performed using logistic regression (odds ratios) for **A** and linear regression (standardized beta values) for **B–D**. Explained variance (*R²*) for the linear models: NIHSS *R*² = 0.53 (**B**), BI *R*² = 0.75 (**C**), UEFM *R*² = 0.70 (**D**). Sample size: *N* = 31. MOD, modularity.

**Figure 4:**
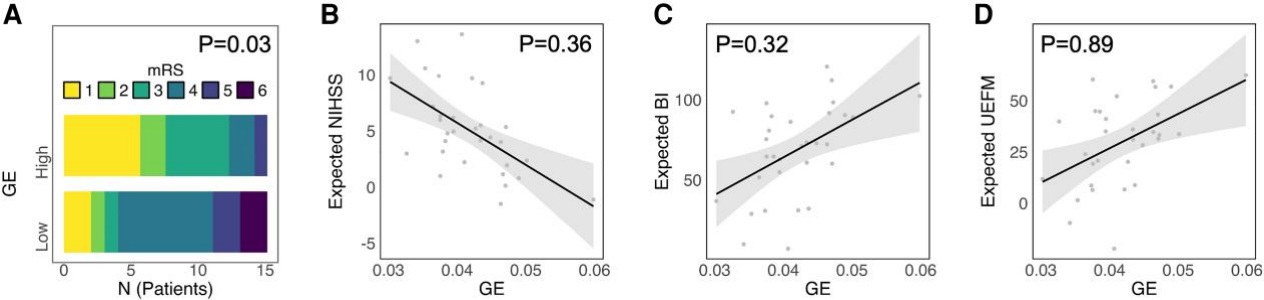
**Outcome correlation with global efficiency.** MRS distribution is illustrated by stacked histograms (**A**) for high or low GE of the structural network after median split dichotomization. For NIHSS (**B**), BI (**C**) and UEFM (**D**), effect plots are given for linear regression analyses with GE with linear fit (grey line), 95% confidence intervals (shaded) and individual point estimates. Statistical analyses were performed using logistic regression (odds ratios) for **A** and linear regression (standardized beta values) for **B–D**. Explained variance (*R²*) for the linear models: NIHSS *R*² = 0.44 (**B**), BI *R*² = 0.73 (**C**), UEFM *R*² = 0.68 (**D**). Sample size: *N* = 31.GE global efficiency.

Network topology–outcome relationships were explored using ordinal logistic and linear regression modelling. Lower MOD was significantly associated with a more favourable outcome, operationalized by lower mRS (*P* < 0.001), lower NIHSS (*P* = 0.02) and higher BI (*P* = 0.01). For UEFM, the association, similar in direction, did not reach the level of significance (*P* = 0.30, Table 2, Fig. 3). For GE, significances were less consistent across outcome measures and were only evident for mRS (*P* = 0.03), but not NIHSS, BI or UEFM anymore (all *P* > 0.32, Table 2, Fig. 4). Figures 3 and 4 give visualizations of the four MOD–outcome and GE–outcome associations. For sensitivity analyses, we recomputed MOD and GE values based on the Brainnetome Atlas.^24^ Statistical findings remained stable, with lower MOD associating with a better outcome in all clinical scores (all *P* < 0.02) except UEFM (*P* = 0.07) and higher GE exhibiting an isolated relation to lower mRS scores at follow-up (*P* = 0.03, see Supplementary Table 1).

**Table 2:** Relationship between structural network topologies at T_1_ and clinical scores at T_2_

	mRS		NIHSS		BI		UEFM	
	OR	*P*	Beta	*P*	Beta	*P*	Beta	*P*
**MOD**	30.41	< 0.001	0.48	0.02	−0.40	0.01	−0.15	0.30
**GE**	0.18	0.03	−0.18	0.36	0.13	0.32	0.02	0.89

Results of ordinal (mRS) and multiple linear (NIHSS, BI, UEFM) regression models associating MOD and GE to four clinical scores mRS, NIHSS, BI and UEFM, corrected for age, log-transformed lesion volume and initial deficit (NIHSS T_1_). Estimated odds ratios (OR) or standardized coefficients (Beta) are given for MOD and GE. *P* values are uncorrected. MOD, modularity; GE, global efficiency.

## Discussion

The main finding of the present study was that network topological estimates of brain reserve, derived from an analysis of contralesional structural brain networks within the first days after stroke, were significantly linked with outcome variability. Specifically, the most consistent association with different outcome scores was detected for network modularity at baseline, an indicator of network segregation: lower modularity was significantly associated with a more favourable outcome with lower levels of global and activity-related disability and neurological symptom burden, independent of the initial deficit, lesion volume and age. In contrast, global efficiency, a measure of network integration irrespective of modular functioning, did not show a similarly consistent relationship with most clinical scores. This result sheds novel light on brain reserve, localizing within the topology of structural networks, as a critical determinant of recovery after stroke.

This finding adds a brain reserve perspective to previous studies reporting time-dependent changes in the topology of ipsilesional and contralesional structural brain networks after stroke. A longitudinal analysis of 30 acute stroke patients reported an exponential increase in modularity of both hemispheres between the first days after the event and 12 months. The ipsilesional increase in modularity over time, most likely due to the loss of long-distance connections,^30^ was associated with more significant clinical deficits at follow-up.^16^ The present data indicate that lower modularity obtained at baseline is linked to more favourable outcomes after stroke. Thus, and on a speculative note, less segregated networks before stroke could show enhanced robustness against network segregation, which disproportionately commences after the event. Interestingly, functional and structural network analyses have demonstrated that already healthy aging without stroke at around 60–70 years and older leads to a gradual increase in network segregation,^31,32^ most likely due to decreases in within-module connectivity and increases in between-module connectivity.^33,34^ Thus, an additional stroke might intensify network segregation during healthy aging, which might finally result in detrimental disconnecting network effects over time.^35^ For instance, one study in chronic stroke patients found that greater network segregation, quantified via modularity, was associated with more severe chronic aphasia.^36^ Consequently, following the concepts of brain reserve capacity in stroke recovery research, lower network modularity might be interpreted as a measure of lower brain age^3^ and greater network reserve characterized by the relative predominance of large-scale, more integrated, less localized or clustered networks. Similar interpretations have been made for cerebellar brain morphometry: larger brain volumes might compensate for post-stroke atrophy.^6^

Network modularity has also been addressed by functional imaging studies and related to inter-subject variability in various therapeutic interventions for healthy aging and after stroke or brain injury.^37^ In contrast to our findings, studies have consistently reported positive associations between modularity and outcome. For instance, higher resting-state modularity was linked to exercise-related gains in executive functioning in older adults,^38^ to better improvement during language therapy in chronic stroke patients,^39^ and training-related cognitive gains in patients with traumatic brain injury.^40^ In subacute stroke patients, one resting-state study reported an early decrease of ipsilesional modularity and a gradual reinstatement of modular network configuration within 1 year, which was related to the amount of recovery.^18^ Thus, the question arises as to why these functional network studies found a positive link between higher modularity and better, but not worse, outcomes. First, one possible explanation might be the difference between brain structure and function. Although functional and structural networks show many common topographic features,^41^ there is only a weak correspondence regarding modular organization.^42^ Structural modules have been reported to be spatially relatively compact and contiguous. Except for some homotopic modules located along the medial wall,^43^ they are usually restricted to a single hemisphere. In contrast, functional network modules were mainly characterized as spatially distributed, comprising distant and homotopic regions,^44,45^ which do not need to be structurally connected. Hence, given corroborating structural analyses over lifespan,^32^ the present data emphasize that structural and functional properties might capture different aspects of network organization. Second, an alternative explanation might affect the difference between intrahemispheric and global assessments of networks. In the present study, we investigated the contralesional network topology to approximate the pre-stroke state of the brain structural networks and, therefore, not the lesioned ipsilesional or global networks. This might explain the different directions of association between modularity increases and favourable outcomes in one functional network analysis in early subacute stroke patients.^18^ Finally, studies are variable regarding the leading domains of deficits, the level of impairment, time after stroke and stroke aetiology. Such factors might also explain differences in directionality between network parameters and behavioural scores.

Network topology was assessed on the contralesional hemisphere to approximate pre-stroke network conditions. Hence, the network–outcome relationships might (i) directly apply to the contralesional hemisphere itself, or as an alternative and potentially more important interpretation (ii), they might hold as a proxy for the ipsilesional hemisphere undergoing the most prominent network effects after stroke.^16^ For the former interpretation, a broad body of literature shows that the contralesional hemisphere is significantly involved in recovery processes after stroke, particularly in more severely impaired patients.^46,47^ One limitation for interpretation (ii) is that functional and structural network analyses in healthy participants reported hemispheric differences, e.g. the left hemisphere is more modular than the right hemisphere.^48^ Further, structural analyses revealed hemisphere and lesion side-specific network modularity alterations in chronic stroke patients.^30^ This might have created a systematic bias. However, in additional sensitivity analyses, we addressed these issues by including the side of the contralesional network (right or left) as an additional covariate in the winning models, and the findings remained stable, which would support interpretation (ii) with contralesional network modularity qualifying as a proxy for the pre-stroke ipsilesional network reserve.

Finally, it was interesting to see that network modularity, but not network efficiency, was most consistently related to different outcome measures after stroke, although the overall directions of associations between GE and the clinical scores would align with the MOD findings. An explanation for this result remains elusive. Similar discrepancies between efficiency and modularity are known from the literature: for instance, Siegel *et al.*^18^ did neither find stroke-related changes in function network efficiency nor reported any associations between efficiency and recovery trajectories. In our previous work, we could link a better outcome to preserved efficiency but not modularity of structural brain networks after the lesion.^9^ Hence, discrepancies between studies in topology–outcome relationships might also arise from the level of analysis, i.e. whether global, bilateral networks or lateralized networks are considered. The present data argue that network reserve, addressed via intact, spared structural connectomes, might be better captured by their modularity than efficiency, and modularity might better grasp the potential of reorganization within the hemisphere than efficiency. Future studies are needed to address this discrepancy between modularity and efficiency regarding brain and network reserve in more detail.

Several significant limitations are worth noting. First, the sample size is relatively small, and larger cohorts are needed to confirm our results. In mRS modelling, we addressed the sample size by using binary dichotomization to increase statistical power and overcome the limitation of potential outliers, which aligns with our previous.^6,7,49^ Second, tractography on the contralesional hemisphere was based on imaging data acquired within the first 2 weeks after stroke to estimate a pre-stroke proxy of the network topology. Although we cannot entirely exclude that stroke-related brain alterations might alter contralesional structural networks already in this early time window, previous analyses in chronic stages of recovery argue against this possibility.^30^ To address this issue, (i) we incorporated the covariate *days of imaging* after stroke which did not alter the significant results. In addition, (ii) we recomputed our analysis in a subset of the severely affected cohort, restricted to stroke patients who underwent imaging within the first week post-stroke. The results remained consistent with our main findings (Supplementary Fig. 1). Finally, we used a cohort of healthy controls (*n* = 42) where we found no differences in network measures of the contralesional hemisphere (Supplementary Fig. 2). Third, follow-up data at T_2_ was not acquired at a common time point. Clinical testing was conducted 3 or 6 months after the event. Including this factor in the winning models also did not change the results. Fourth, our cohort consisted only of patients with supratentorial stroke lesions. The dependency of network integration and segregation might play a different role in patients with infratentorial stroke lesions.^50^ Fifth, in agreement with our previous study,^9^ the structural connectome was computed based on the *AAL116* atlas. We re-computed our results for sensitivity analyses by applying the Brainnetome Atlas, and the findings remained stable (Supplementary Table 1). Finally, clinical scores considered in this work are dominated by features of the motor domain. Alternative outcome measures, particularly in the cognitive or language domains, might call for alternative reference networks and alter the present results.

## Supplementary Material

fcaf456_Supplementary_Data

## Data Availability

Data will be made available by the authors upon reasonable request.
