# Supplementary material for: The structural reserve of brain networks influences outcomes after a stroke
Source: Brain Commun. 2025 Nov 20;8(1):fcaf456. doi: 10.1093/braincomms/fcaf456 (PMC12816807; doi:10.1093/braincomms/fcaf456)
Supplement: fcaf456_Supplementary_Data [file fcaf456_supplementary_data.docx]

**Supplementary Materials**

**The structural reserve of brain networks influences outcomes after a stroke**

Lukas Frontzkowski^1,4,5#^, Tim J. Hunze^1#^, Winifried Backhaus^1^,

Marlene Bönstrup^1,2^, Christian Gerloff^1^, Bastian Cheng^1^, Götz Thomalla^1^, Benedikt M. Frey^1^, Paweł P. Wróbel^1^, Hanna Braaß^1^, Philipp J. Koch^3^, Focko L. Higgen^1^, Fanny Quandt^1^, Robert Schulz^1*^

^1^ Department of Neurology, University Medical Center Hamburg-Eppendorf, Hamburg, Germany

^2^ Department of Neurology, University Medical Center Frankfurt, Frankfurt, Germany

^3^ Department of Neurology, University Medical Center Lübeck, Lübeck, Germany

^4^Department of Nuclear Medicine, University Hospital, LMU, Munich, Germany

^5^Institute for Stroke and Dementia Research (ISD), LMU, Munich, Germany

|  | mRS |  | NIHSS |  | BI |  | UEFM |  |
| --- | --- | --- | --- | --- | --- | --- | --- | --- |
|  | OR | P | Beta | P | Beta | P | Beta | P |
| MOD | 15.80 | 0.001 | 0.56 | 0.01 | -0.34 | 0.02 | -0.28 | 0.07 |
| GE | 0.18 | 0.03 | -0.09 | 0.63 | 0.03 | 0.79 | 0.03 | 0.83 |

**Supplementary Table 1 | Relationship between structural network topologies at T_1_ and clinical scores at T_2_ based on Brainnetome atlas data**

Results of ordinal (mRS) and multiple linear (NIHSS, BI, UEFM) regression models associating MOD and GE derived from the Brainnetome atlas to clinical scores, corrected for age, log-transformed lesion volume, and initial deficit (NIHSS T_1_). Estimated odds ratios (OR) or standardized coefficients (Beta) are given for MOD and GE. P values are uncorrected. MOD modularity. GE global efficiency.

**Supplementary Figure 1 | Outcome correlation with network modularity for stroke patients with imaging within first week post-stroke (n=26).**

MRS distribution is illustrated by stacked histograms (**A**) for high or low MOD of the structural network after median split dichotomization, with group differences evaluated using logistic regression. For NIHSS (**B**), BI (**C**), and UEFM (**D**), effect plots are given for linear regression analyses with MOD with linear fit (grey line), 95% confidence intervals (shaded), and individual point estimates. MOD modularity. Statistical analyses were performed using logistic regression (odds ratios) for A and linear regression (standardized beta values) for B-D. Explained variance (*R²*) for the linear models: NIHSS *R² = 0.53* (B), BI *R² = 0.63* (C), UEFM *R² = 0.75* (D). Sample size: N = 31. MOD modularity.


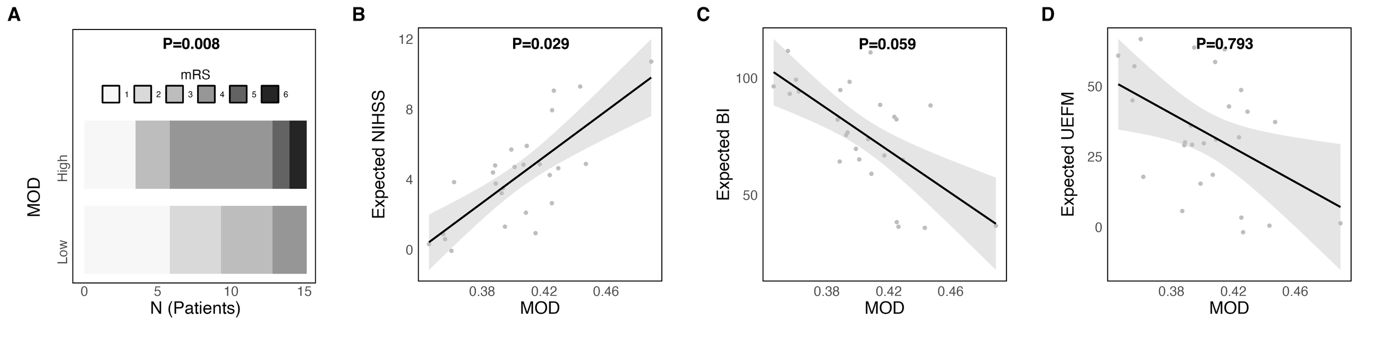


*
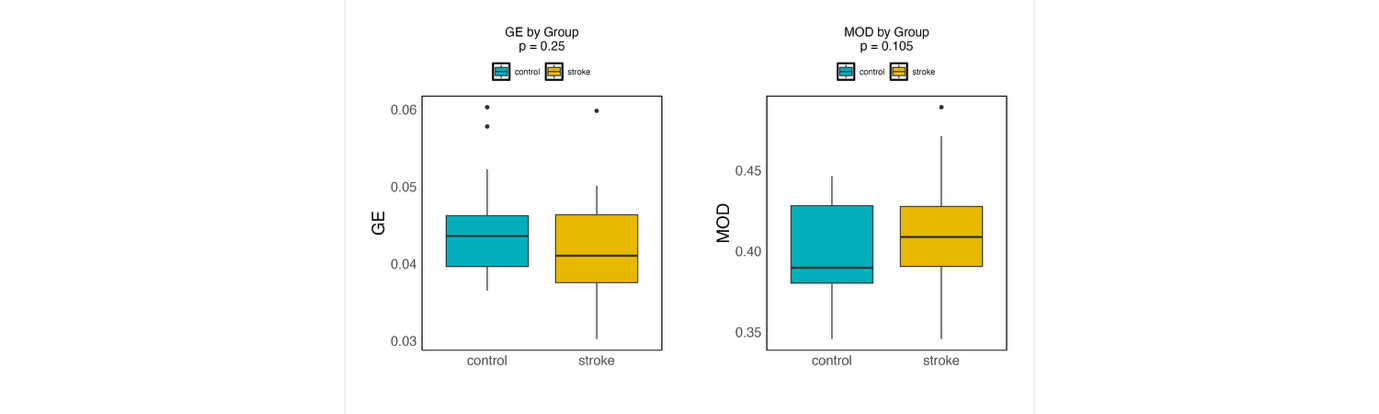
*

**Supplementary Figure 2 | Results of ANOVA analysis.**

Contralesional Network Properties were compared between stroke patients (N = 31) and a cohort of healthy controls (N = 42). Models were corrected for age, sex and hemispheric dominance. GE - Global Efficiency, MOD - Modularity.
